# Clinical evaluation of a novel intravaginal probiotic product on uterine health in postpartum dairy cows

**DOI:** 10.3389/fvets.2026.1821620

**Published:** 2026-07-14

**Authors:** Zsóka Várhidi, György Csikó, Orsolya Palócz, Balázs Erdélyi, Péter Sátorhelyi, Dóra Sulyok, Ferenc Zolnai, Mikolt Bakony, Viktor Jurkovich

**Affiliations:** 1 Department of Animal Hygiene, Herd Health and Mobile Clinic, University of Veterinary Medicine, Budapest, Hungary; 2 Department of Pharmacology and Toxicology, University of Veterinary Medicine, Budapest, Hungary; 3 Fermentia Microbiological Ltd., Budapest, Hungary; 4 Enyingi Agricultural Ltd., Enying, Hungary; 5 Centre for Translational Medicine, Semmelweis University, Budapest, Hungary; 6 Centre for Animal Welfare, University of Veterinary Medicine, Budapest, Hungary

**Keywords:** dairy cattle, intravaginal treatment, metritis prevention, probiotics, reproductive performance, uterine health

## Abstract

Uterine disease, including metritis, affects the majority of dairy cattle herds and significantly reduces both reproductive performance and milk production. Generally applied treatment protocols for metritis include antimicrobials; however, the spread of antimicrobial resistance has increased the need for alternative prevention and treatment methods in recent years. This study aims to evaluate the clinical efficacy of a novel intravaginal probiotic product containing *Brevibacillus agri* and five lactic acid bacteria species for the prevention of uterine disease in multiparous Holstein-Friesian cows. In total, 453 cows participated in the study on two different commercial dairy farms. The treatment protocol included one prepartum and two postpartum treatments with the product. A non-significant tendency toward lower vaginal discharge scores was observed in treated cows across both farms (OR = 0.59, *p* = 0.186). There were no significant changes in rectal temperature, blood BHB and serum iCa concentrations, fetal membrane retention, the number of services/pregnancy, or the length of the service period due to treatment. In conclusion, further investigations are necessary to formulate a product that can be successfully applied to dairy cattle farms to improve uterine health.

## 1 Introduction

Uterine disease is a major challenge for most commercial dairy cattle farms. It affects approximately half of the dairy population and significantly reduces reproductive performance and milk production (1). On large-scale cattle farms, most premature culling happens due to reproductive issues (30% of all premature disposals) (2–5). Uterine disease includes metritis, clinical and subclinical endometritis, and pyometra. Metritis is defined as the inflammation of the uterine wall within the first 21 days of calving. Clinical signs include watery, red-brown uterine discharge with a fetid odour, an enlarged and flaccid uterus, and, in more severe cases, fever, inappetence, lower milk yield, and lethargy (6, 7). Generally, treatment protocols for metritis include a combination of antimicrobials, non-steroidal anti-inflammatory drugs, and hormones, including uterotonics (8). According to a recent Central European study, antimicrobials and hormones account for the two largest shares of veterinary drug costs on commercial dairy cattle farms, and reproductive issues account for the second-largest share, following mastitis (9).

The spread of antimicrobial resistance shifted scientific interest towards alternative treatment and prevention methods in recent years. Probiotics gained special attention within this category. Probiotics can potentially reduce the dependence on antimicrobial agents. However, practitioners are often unsure about the proper dosage, the optimal timing of treatment, and potential side effects, which makes them less likely to use these products daily (10). So far, the most intensely researched group of bacteria for a potential intravaginal probiotic product has been lactic acid bacteria. *In vitro* studies described beneficial characteristics of several bacterial strains, but *in vivo* studies have been inconclusive (11). Direct, strain-level persistence data for cattle or other ruminants after intravaginal administration are largely not reported or not detailed in studies on probiotic administration. Several bovine trials assessed clinical and microbiological outcomes. Still, they did not report validated, strain-specific tracking of the administered probiotics over time, so the duration of colonization beyond days to a few weeks in cattle is not well established from these studies (12–14). Where studies evaluated cultivable LAB counts after treatment, they often reported increases in lactobacilli or LAB abundance in the vaginal milieu. Still, these measures do not prove that the administered strain(s) persisted specifically, rather than transiently increased indigenous LAB or non-tracked probiotic cells (15, 16). Commonly described modes of action of probiotics include modification of initial microbial load and diversity by colonization or biofilm formation (11, 17). Murine and human data show that Lactobacilli exhibit short, transient persistence after focal administration (18–20). To market a reliable and effective veterinary probiotic product, further investigation is necessary.

This research aimed to evaluate the clinical efficacy of the novel intravaginal probiotic product (21) for preventing and controlling postpartum uterine diseases under field conditions in a large number of cows. Multiple parameters were included in the analysis to assess the effects of the product on uterine health, involution, occurrence of metritis, and signs of inflammation. The ultimate goal was to develop a product that can be easily integrated into daily farm protocols and has the potential to reduce antimicrobial use while simultaneously improving reproductive performance.

## 2 Materials and methods

### 2.1 Sample size calculations, screening, and enrolment

The study was ethically approved by the Pest County Government Office (experimental permit number: PE/EA/00775-4/2023).

The study was conducted on two large-scale dairy cattle farms in Hungary. Table 1 shows the basic data of both farms included in the study.

**Table 1:** Basic data of farms involved in the study.

Item	Farm A	Farm B
Total number of cows	1,700	511
Barn, before calving	Straw-bedded yard	Straw-bedded yard
Barn after calving	Freestall, rubber mattress	Straw-bedded yard
Milking system	Parallel parlor, twice daily	Parallel parlor, twice daily
Daily milk yield (kg)	32.5	32.6
Calving interval (days)	407	401
Calving to first insemination (days)	59	66
Calving to pregnancy diagnosis (days)	127	117
Number of services/pregnancy	3.3	2.5
Productivity	71.2%	81.3%

Sample size was determined using the sample size calculator at https://clincalc.com/stats/samplesize.aspx. For dichotomous variables, we calculated the proportion of healthy vaginal discharge (VD0) as an example (80% in the treated and 68% in the untreated groups), which is an interpretable and acceptable difference. In this way, the required sample size was 209 per group, a total of 418 (alpha: 0.05; beta: 0.2; power: 0.8). For the continuous variables, we calculated with the number of artificial inseminations per pregnancy as an example (2.8 in the treated and 3.5 in the untreated group as an interpretable and acceptable difference). In this way, the required sample size was 200 per group, a total of 400 (alpha: 0.05; beta: 0.2; power: 0.8). In total, 453 multiparous (parity ≥ 2) Holstein-Friesian cows were enrolled across both farms over an approximately three-month period, with the daily enrolment rate determined by the number of calvings at each site. Eligible cows were identified 21 days before their expected calving date, at the time of transfer from the dry cow barn to the pre-calving barn.

Eligibility was confirmed by clinical assessment, including body condition scoring and locomotion scoring (22, 23). Cows were allocated to the experimental (EXP) or control (CO) group based on the parity of their ear tag number: cows with odd ear tag numbers were assigned to the EXP group and those with even numbers to the CO group. This systematic allocation method was applied consistently across both farms.

**Figure 1:**
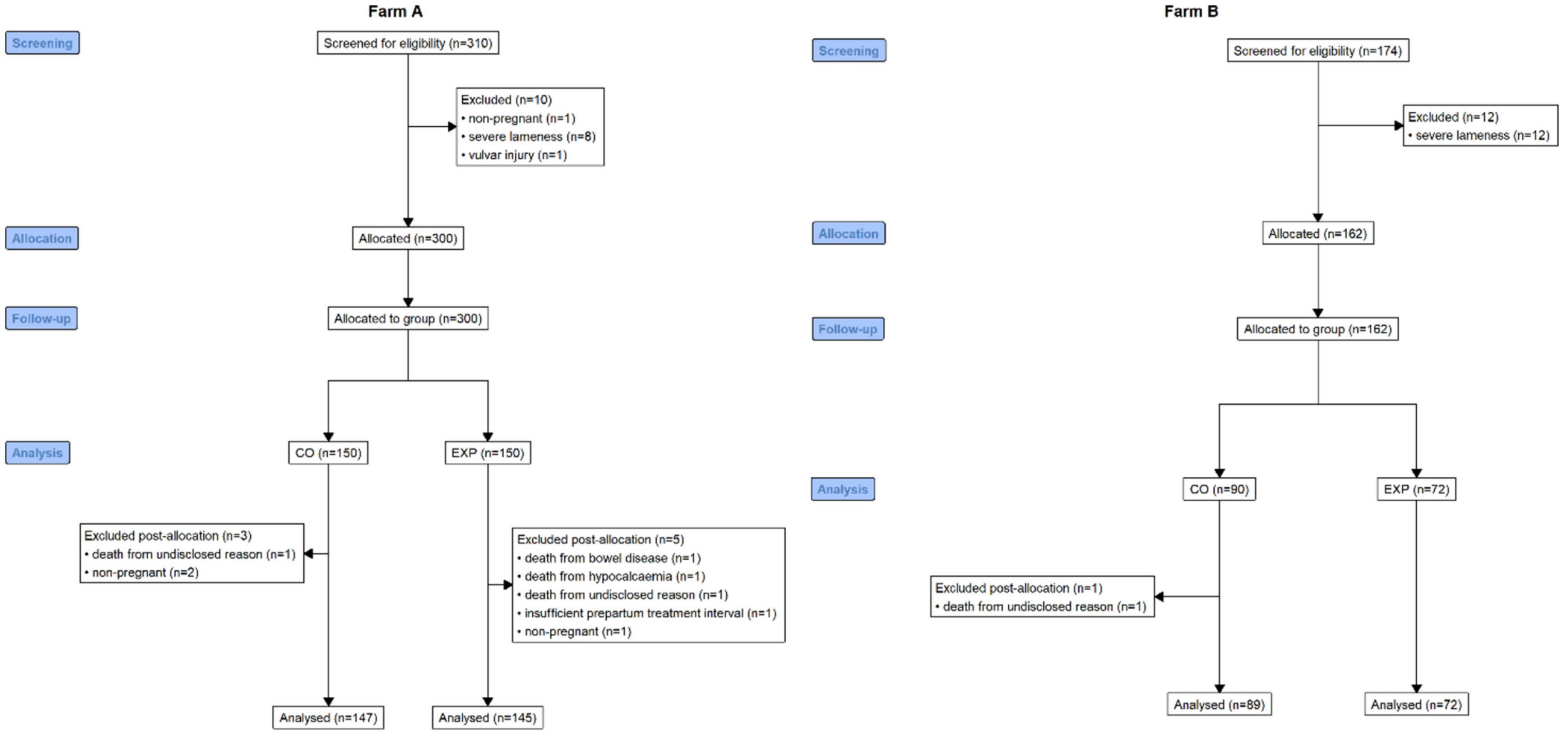
Participant flow diagram. Multiparous Holstein-Friesian cows were screened for eligibility 21 days before expected calving during an approximately three-month enrolment period on two commercial dairy farms. Eligible cows were allocated to the experimental (EXP) or control (CO) group based on the parity of their ear tag number. Post-allocation exclusions occurred before the first postpartum examination. All remaining cows were included in the analysis for as long as data were available.

The small imbalance in group sizes observed on Farm B (EXP *n* = 72, CO *n* = 89) reflects the unequal distribution of odd and even ear tag numbers among the cows that calved during the enrolment period, compounded by unexpected losses from the experimental group, and was not the result of a procedural deviation. The flow of animals through the study is presented in Figure 1.

### 2.2 Treatment and examination protocol

The intravaginal probiotic product was administered on three occasions: at enrolment and randomization (21 days before the expected calving date), and at 3–5 and 7–9 days postpartum. The timing of treatments was designed to align with the routine management procedures of both farms, minimizing the number of additional animal-handling procedures beyond those already scheduled.

The first treatment was administered at the time of transfer from the dry cow pen to the pre-calving pen, which occurs routinely at approximately 21 days before expected calving on both farms. The second and third treatments coincided with the scheduled postpartum involution examinations at days 3–5 and 7–9, respectively. The administration procedure was as follows: the vulva was cleaned with Iodine and 70% alcohol, and afterward, 10 mL of the product (Table 2) was injected into the vagina near the cervix using a sterile catheter. Utmost care was taken so that disinfectant did not enter the vagina, and the process was as swift as possible.

**Table 2:** Composition of the probiotic product.

Component	Composition
Carrier	1.5% HEC, 1.0% dextrose in PBS buffer; pH = 6.0
Prebiotic component	1.0% inulin
Probiotic components	*Limosilactobacillus fermentum* 5×10^8^ CFU/dose, *Lactiplantibacillus plantarum* 5×10^8^ CFU/dose, *Limosilactobacillus reuteri* 5×10^8^ CFU/dose, *Lacticaseibacillus rhamnosus* 5×10^8^ CFU/dose, *Pediococcus pentosaceus* 5×10^8^ CFU/dose, *Brevibacillus agri* 5×10^8^ CFU/dose

Postpartum examinations followed the standard clinical protocol of both farms and were performed at five time points: within 24–48 h of calving, and at days 3–5, 7–9, 14, and 21 postpartum.

At 24–48 h, passage of fetal membranes was recorded. Blood samples were collected 1 day after calving in the morning, between 8:00 and 10:00, from the coccygeal vein into lithium-heparin-containing Vacuette tubes (Greiner Bio-One International GmbH, Kremsmünster, Austria). Ionized calcium was measured from fresh whole blood samples using the LAQUAtwin Ca-11 blood iCa device (HORIBA Advanced Techno. Co. Ltd., Kyoto, Japan) (24).

At days 3–5, 7–9, and 14, examinations included rectal palpation to assess uterine size and consistency, uterine massage to evaluate the volume and character of vaginal discharge, and measurement of rectal temperature. Vaginal discharge was scored on a four-point scale (VD 0–3) as described below. At days 7–9, blood samples were taken for BHB concentration measurements. Blood samples were collected from the coccygeal vein into 9 mL Li-heparin-containing Vacuette tubes (Greiner Bio-One International GmbH, Kremsmünster, Austria). Samples were measured immediately with a handheld BHB meter (BHB-Check, TaiDoc Technology Co., New Taipei City, Taiwan) (25).

At day 21 postpartum, body condition and locomotion were re-evaluated, and a final involution examination was performed.

### 2.3 Clinical endpoints of uterine health and reproductive success

The primary biomarker of uterine health status was the quality of vaginal discharge. Vaginal discharge (VD) was scored during involution examinations, using the following scoring system based on pre-existing literature (6, 26): VD-0 = no or clear discharge; VD-1 = clear discharge with flakes of pus; VD-2 = discharge containing less than 50% pus, not watery, no fetid odor; VD-3 = watery discharge containing more than 50% pus, dark red or brown color, fetid odor. A score of 0 was considered physiological, while scores 1–3 were considered abnormal.

Involutional complications, including the incidence of retained fetal membranes and fever (rectal temperature >39.5 °C), were considered as secondary endpoints to uterine health. Fever was treated as an independent indicator of systemic inflammatory response, given that it may precede or occur independently of changes in vaginal discharge consistency in cows with postpartum uterine infection. Although a composite outcome incorporating both vaginal discharge score and rectal temperature would have provided a more complete clinical picture of uterine disease severity, the two parameters were analysed independently in the present study, which is acknowledged as a limitation.

Examinations and treatments carried out during the study period are summarized in Table 3.

**Table 3:** Examinations and treatments during the study.

Time	Examination	Treatment
21 days prepartum	Body condition scoring, locomotion scoring	EXP group 1st probiotic treatment
48 h postpartum	Fetal membranes check, iCa measurement	
3–5 days postpartum	Involution examination (VD scoring), rectal temperature	EXP group 2nd probiotic treatment
7–9 days postpartum	Involution examination (VD scoring), rectal temperature, BHB measurement	EXP group 3rd probiotic treatment
14th day postpartum	Involution examination (VD scoring)	
21st day postpartum	Involution examination (VD scoring)	

EXP: experimental group; VD: vaginal discharge; BHB: beta-hydroxybutyrate; iCa: ionized calcium.

Reproductive data, including the number of services per pregnancy and the length of the period from calving to first insemination, were collected from the farm database.

### 2.4 Statistical analysis

Association between postpartum indicators and the treatment were explored using exploratory visualizations and hypothesis testing. Randomization success was confirmed by comparing prepartum parameters.

To address the ordinal nature of VD scores and to model the full longitudinal trajectory across all four examination time points, a cumulative link mixed model (CLMM) was fitted. Cumulative link mixed models estimate the effect of predictors on an ordered outcome—in this case VD scores ranging from 0 to 3—by modelling the probability of observing each score category and expressing the treatment effect as an odds ratio, which reflects how much more or less likely treated cows were to have a higher VD score compared to controls, while accounting for the fact that repeated measurements taken from the same cow are not independent of one another. VD score (0–3) was the ordered outcome. Group (EXP vs. CO), time point (days 3–5, 7–9, 14, and 21 postpartum, modeled as a factor), and Farm were included as fixed effects. Cow identity (ID) was included as a random intercept to account for repeated measures on the same animal. Time was modeled as a factor rather than a continuous variable, as a likelihood ratio test confirmed that the linear time assumption was significantly violated [χ^2^(2) = 39.25, *p* < 0.001], consistent with the non-linear postpartum trajectory of VD scores. The proportional odds assumption was evaluated using the nominal test and scale test on the equivalent marginal model (clm, without random effect), as these tests are not available for mixed models in the current implementation of the *ordinal* package. The assumption was satisfied for the treatment group effect (nominal test: *p* = 0.874), but was violated for time and farm, consistent with known variation in discharge severity across time points and between farms. As the key predictor of interest satisfied the assumption, the proportional odds CLMM was retained as the primary model.

To complement the trajectory analysis, the cumulative discharge burden for each cow was quantified as the area under the VD score-time curve (AUC), calculated using the trapezoidal rule across all available time points. Group differences in AUC were assessed using a Wilcoxon rank-sum test stratified by Farm, as the AUC distribution was heavily zero-inflated (63% of cows had AUC = 0), precluding the use of linear models.

Regarding confounding variables, including the proportion of animals with fever, hyperketonaemia, and hypocalcaemia, respectively, the control and EXP groups were compared using Fisher’s exact test separately for each farm.

For numerical variables, including number of services per pregancy, length of service period, means were compared between the two groups using a linear model. Differences in the management of the periparturient period between farms were accounted for by including Farm as a block effect and Farm x treatment interaction term. The interaction term was removed if it was non-significant.

Significance level was set at *p* < 0.05. All data visualization analyses were performed in R version 4.4.3 using *dplyr*, *ggplot2*, *consort*, *stats*, *ordinal* and *coin* packages (27).

## 3 Results

The number of animals screened, allocated, and analysed on each farm is summarised in Figure 1. The following prepartum parameters were used to confirm randomization: number of lactations, body condition score, and locomotion score (Table 4). There were no significant differences between the control and EXP groups for any of the tested parameters, indicating successful randomization (*p* > 0.05). The actual timing of enrolment/prepartum treatment in relation to calving for control and experimental cows was 23.4 ± 6.6 days and 22.3 ± 6.3 days on Farm A, and 19.9 ± 3.2 days and 20.8 ± 4.2 days on Farm B, respectively.

**Table 4:** Mean and standard deviation of basic prepartum data.

Farm	Group	Number of cows	Number of lactations	Body condition score	Locomotion score
A	EXP	145	2.7 ± 0.9	3.5 ± 0.5	1.7 ± 1.0
	Control	147	2.8 ± 1.0	3.5 ± 0.5	1.7 ± 0.9
B	EXP	72	2.2 ± 1.1	3.1 ± 0.3	1.6 ± 0.8
	Control	89	2.3 ± 1.1	3.1 ± 0.4	1.6 ± 0.9

EXP: experimental group.

**Figure 2:**
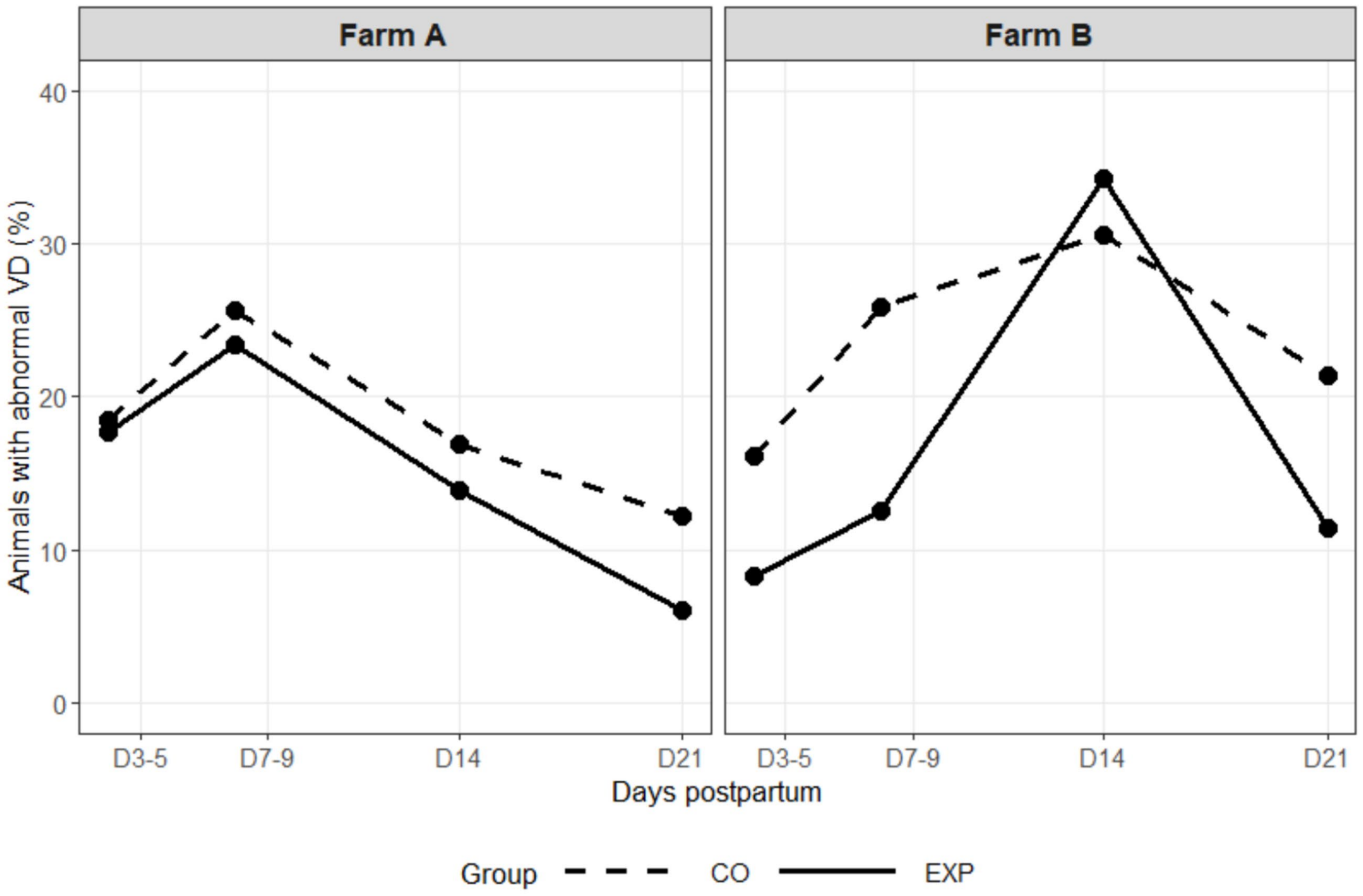
Proportion of cows with abnormal discharge on days 3–5, 7–9, 14, and 21 postpartum.

The proportion of animals for each VD score at each examination time is displayed per farm in Table 5. To better visualize the non-linear association between time and the distribution of VD scores, which helps interpret the ordinal regression model’s results, Figure 2 shows the proportion of animals with abnormal vaginal discharge (VD 1–3) over time.

**Table 5:** Vaginal discharge scores on days 3–5, 7–9, 14, and 21 postpartum.

Farm	Group	Physiological	Abnormal			Physiological	Abnormal		
		0 (%)	1 (%)	2 (%)	3 (%)	0 (%)	1 (%)	2 (%)	3 (%)
		Days 3–5				Days 7–9			
A	EXP (*n* = 145)	82	7	3	8	77	11	4	8
	Control (*n* = 147)	82	8	2	8	74	7	8	11
B	EXP (*n* = 72)	92	1	1	6	87	0	3	10
	Control (*n* = 89)	94	2	1	13	74	2	7	17
		Day 14				Day 21			
A	EXP (*n* = 145)	86	2	9	3	94	4	2	0
	Control (*n* = 147)	83	9	6	2	88	7	4	1
B	EXP (*n* = 72)	66	4	24	6	89	4	6	1
	Control (*n* = 89)	70	2	20	8	79	7	13	1

EXP: experimental group.

The cumulative model fitted 1744 observations from 447 cows. Convergence was satisfactory (max. Gradient = 1.14 × 10^−4^). Individual cow-level variation in VD scores was large (random intercept SD = 3.4), indicating that repeated measures on the same animal were strongly correlated. Treated cows had approximately 40% lower odds of being in a higher VD category than controls, although this difference was not statistically significant (OR = 0.591, 95% CI: 0.27–1.28, *p* = 0.1857). Regarding the temporal changes, in reference to days 3–5, VD scores were significantly worse at days 7–9 (OR = 2.27, 95%CI: 1.48–3.49, *p* = 0.0002). This reflects the well-known peak of uterine discharge in the first week postpartum. By day 14, scores had begun to decline but remained numerically above the day 3 level, though not significantly (OR = 1.386, 95%CI: 0.89–2.14, *p* = 0.1426). By day 21, VD scores were significantly lower than at day 3, odds of a higher category were 0.34 times those at day 3 (95% CI: 0.21–0.57, *p* < 0.0001), indicating substantial resolution of discharge by the end of the observation period. The numerical trend toward higher VD scores at FarmB was not significant after accounting for treatment and time (OR: 1.2, 95% CI: 0.53–2.3, *p* = 0.6536). The threshold coefficients (cumulative log-odds of 3.61, 4.47, and 6.04 for the 0|1, 1|2, and 2|3 thresholds, respectively) reflected the high baseline proportion of physiological discharge in both groups.

The cumulative discharge burden (AUC) did not differ significantly between groups (Wilcoxon test stratified by Farm: *Z* = 1.02, *p* = 0.309). The proportion of cows with any abnormal vaginal discharge over the study period was 38.7% in controls and 34.8% in treated cows. These findings were consistent with the ordinal mixed model, which showed a non-significant tendency toward lower VD scores in treated cows (OR = 0.59, *p* = 0.186).

Regarding fetal membrane retention, the number of services/pregnancy and the length of service period, there was no significant difference due to the treatment between the control and the EXP groups on any of the farms (Table 6). There were no significant differences between the groups in the treatment effect on the proportion of animals with fever (rectal temperature >39.5 °C; Table 7), in the proportions of cows with physiological or abnormal (hypocalcaemia) iCa concentrations between the groups (Table 8), or in the proportions of cows with physiological or abnormal (ketosis) BHB levels (Table 9).

**Table 6:** Retained fetal membranes, number of services/pregnancy, and length of calving to first insemination during the postpartum period.

Farm	Group	Retained fetal membranes (%)	Number of services/Pregnancies	Calving to first insemination (days)
A	EXP (*n* = 145)	5.5	3.5 ± 2.2	59 ± 7
	Control (*n* = 147)	4.8	3.0 ± 1.9	58 ± 7
B	EXP (*n* = 72)	9.7	2.8 ± 2.0	67 ± 5
	Control (*n* = 89)	12.3	3.0 ± 2.2	67 ± 4

EXP: experimental group.

**Table 7:** Rectal temperature on days 3–5 and 7–9 postpartum.

Farm	Group	Days 3–5		Days 7–9	
		Physiological (%)	Fever (%)	Physiological (%)	Fever (%)
A	EXP (*n* = 145)	99.3	0.7	98.0	2.0
	Control (*n* = 147)	100.0	0.0	98.0	2.0
B	EXP (*n* = 72)	98.5	1.5	98.0	2.0
	Control (*n* = 89)	95.0	5.0	86.0	14.0

Rectal temperature >39.5°C was considered high (fever); EXP: experimental group.

**Table 8:** Ionized calcium concentration within 48 h after calving.

Farm	Group	Physiological (%)	Hypocalcemia (%)
A	EXP (*n* = 145)	48	52
	Control (*n* = 147)	43	57
B	EXP (*n* = 72)	15	85
	Control (*n* = 89)	17	83

Ionized Ca was considered physiological when ≥1.05 mmol/L, EXP: experimental group.

**Table 9:** Blood beta-hydroxybutyrate concentration on days 7–9.

Farm	Group	Physiological (%)	Hyperketonemia (%)
A	EXP (*n* = 145)	65	35
	Control (*n* = 147)	65	35
B	EXP (*n* = 72)	82	18
	Control (*n* = 89)	78	22

Blood beta-hydroxybutyrate concentration was considered physiological under 1.1 mmol/L; EXP: experimental group.

## 4 Discussion

The results of our previous study on the safety of a novel intravaginal product enabled a clinical efficacy trial to assess the *in vivo* characteristics of potential probiotic strains (21). The current study was conducted on two large-scale commercial dairy farms in Hungary, involving a total of 453 Holstein-Friesian cows. Our tested product contained a larger amount of carrier (10 mL) than other studies, which generally used 1–4 mL (28–30). The timing of treatments, application method, and CFU dosage were similar to those of other study groups (12, 13, 17, 18, 28–30). Compared to other researchers, who suggest using their product within a few hours, which is not feasible in field conditions, this product has a shelf life of 2 weeks when stored in a refrigerator. Our tested product included *Brevibacillus agri* isolated from healthy cows’ vaginal flora (31) as the main probiotic agent and five lactobacilli to support the probiotic effect (*Limosilactobacillus fermentum, Lactiplantibacillus plantarum, Limosilactobacillus reuteri, Lacticaseibacillus rhamnosus, Pediococcus pentosaceus*) (21). According to our previous study, no lactobacilli were detected in the healthy vaginal flora of dairy cows, and the vaginal pH was around 7 (31). We used lactobacilli in the formulation because others had reported their use (12, 28–30), and we speculated that lactobacilli would lower vaginal pH, eliminate harmful bacteria (32), and allow *Brevibacillus agri* to colonize. Our previous study indicated this, as brevibacilli were still detectable 2 weeks after application, whereas lactobacilli were not (21).

Although treated cows consistently tended to have lower VD scores, the present study did not demonstrate a statistically significant effect of intravaginal probiotic treatment on vaginal discharge scores over the observation period. No significant treatment effects were detected for any of the secondary outcome parameters, including rectal temperature, ionized calcium concentration, blood beta-hydroxybutyrate, fetal membrane retention, or reproductive performance indicators.

The consistent direction of the treatment effect across all outcomes and the observed numerical differences in VD scores, particularly on Farm B at days 7–9 postpartum, suggest that the product may exert a modest biological effect in the period when metritis most commonly occurs (6). Whether this tendency reflects a genuine, clinically meaningful effect of the probiotic treatment warrants investigation in future trials with larger sample sizes and a more homogeneous study population.

A notable feature of the present study was the substantial heterogeneity between the two farms in baseline uterine health status, housing conditions, and the prevalence of metabolic disease. Limitations in measuring statistically significant changes might include farm-specific characteristics such as vaginal microbiota composition, pathogen burden, hygiene, or calving management. It leads us to the interesting assumption that in herds with a high overall disease burden and predisposing factors—including metabolic disorders, hygiene conditions, and calving management—that are unfavorable, the capacity of a locally administered intravaginal product to counteract the prevailing systemic and environmental pressures on uterine health is likely limited. On the other hand, in well-managed herds where the postpartum uterine health status is already satisfactory, the scope for detecting an additional beneficial effect of any supplementary intervention is inherently narrow.

Alternatively, farm-specific treatment protocols might be necessary, taking into account the farm’s postpartum uterine health status. For example, on farms, where metritis is likely to occur within the first few days after calving, an additional treatment could be inserted into the protocol in the first 24–48 h postpartum, and/or the first (prepartum) treatment could be done closer to the expected time of calving, e.g., 14 days precalving.

Although farm-level differences were accounted for in the present study, they represent sources of biological variation that were likely far more influential on postpartum uterine health outcomes than any effect attributable to the treatment itself. Future trials should consider conducting studies within single, well-characterised herds to reduce this background heterogeneity and improve the sensitivity of the efficacy assessment.

Other studies have also reported conflicting results. The differences might be due to the probiotic strains used or the length and frequency of application, which we expand upon in the followings.

*Lactiplantibacillus plantarum* was previously tested in a murine model of bovine endometritis induced by *Escherichia coli*, in which daily treatment for six consecutive days significantly reduced inflammatory cell infiltration and improved histopathological lesions compared with untreated controls (14). Studies using more frequent administration schedules have generally reported more favourable outcomes. Deng et al. (12) treated cows once weekly from weeks −2 and −1 prepartum, with or without an additional postpartum treatment, and found lower incidence of metritis and uterine infection in both treatment groups, although effects on inflammatory markers were inconclusive. Intravaginal lactic acid bacteria treatment also showed inconsistent effects on milk yield and feed conversion, with benefits observed in multiparous but not primiparous cows (13, 28). Ametaj et al. (28) reported a significantly lower incidence of purulent vaginal discharge and reduced haptoglobin concentrations in cows treated weekly from 2 weeks prepartum through 4 weeks postpartum, without effect on pregnancy rate. A similarly frequent protocol applied from week −2 through week +4 postpartum resulted in earlier uterine involution, fewer cases of abnormal vaginal discharge, fewer services per pregnancy, and a shorter service period (29). A prepartum-only protocol—twice weekly from week −3 with no postpartum treatment—reduced metritis and endometritis prevalence on one of two farms but showed no overall significant effect (30). Intravaginal treatment with a mixture of Lacticaseibacillus rhamnosus, *Pediococcus acidilactici*, and Limosilactobacillus reuteri applied twice weekly from week −3 prepartum reduced metritis prevalence, whereas a single intrauterine dose postpartum did not (31). The same bacterial combination applied at days −20 and −10 prepartum improved vaginal and uterine microbiota but did not reduce metritis prevalence or improve conception rate (32). As demonstrated by the studies above, the effects of intravaginal or intrauterine probiotic treatment are often inconclusive. Postpartum uterine diseases are multifactorial, with several risk factors contributing to them (33). In addition to bacterial causes, metabolic problems such as periparturient hypocalcemia and ketosis further elevate the risk of uterine infections (33). Periparturient hypocalcemia weakens the immune system and impairs its ability to respond to uterine infections (34, 35). Elevated blood ketone concentrations resulting from negative energy balance also suppress the immune response, making cows more susceptible to infections, including those in the uterus (36, 37). Interestingly, on Farm B, the proportions of retained fetal membranes and animals with fever and ketosis were lower in the EXP group, but this was only a numerical, not statistical, difference. Otherwise, there was no difference in the proportions of fever, ketosis, or hypocalcemia between the treated and control groups in our study. It should be noted that the proportion of hypocalcemia was high on both farms.

The present study adds to a literature that is difficult to synthesise due to heterogeneous protocols, small sample sizes, and a likely under-representation of null findings. We consider the transparent reporting of inconclusive results from a large field trial to be a necessary complement to the existing evidence base, and hope it will contribute to more realistic expectations regarding the clinical efficacy of intravaginal probiotic products under commercial dairy farm conditions.

## 5 Limitations

Several limitations of the present study must be acknowledged.

The control group received no placebo treatment. Although the administration procedure was performed in the shortest possible time frame and disinfection was carefully limited to the vulvar region, the contribution of the procedure per se to any observed differences cannot be excluded, and future trials should include a vehicle-only control arm.

Complete blinding of personnel was not feasible under field conditions. Due to the extended enrolment period, the large number of animals, and the three-occasion treatment protocol, farm workers and examiners were aware of group allocation. Although VD scoring was performed by a single examiner per cow with a second opinion sought in uncertain cases, observer bias cannot be ruled out, and inter-rater reliability was not formally assessed.

The per-farm analysis was not pre-specified in the original study protocol. The decision to examine farms separately was made during the analysis phase, prompted by the observation that the two farms differed substantially in housing conditions, baseline metabolic disease prevalence, and overall postpartum uterine health status. By that point, enrolment had already been completed, and the sample size available on Farm B was constrained by the animal resources at that site. A prospective per-farm analysis with adequate statistical power at each site would have required a substantially larger study than was feasible within the resource constraints of the present trial.

The composition of vaginal microbiota was not analysed before or after treatment, and no inflammatory markers were measured from blood at all time points. These data would have provided mechanistic insight into the product’s mode of action and allowed assessment of whether *Brevibacillus agri* colonisation occurred as intended.

Concomitant antibiotic use during the study period was not prohibited, as farms were permitted to follow their own treatment protocols throughout the trial. In line with current antimicrobial resistance prevention regulations applicable to all Hungarian dairy cattle farms, antibiotic use was kept to a minimum at both sites. Treatment of inadequate uterine involution followed standard farm protocols, consisting primarily of uterine massage, prostaglandin F2α, and non-steroidal anti-inflammatory drugs, with antibiotic administration reserved as a last resort at the discretion of the farm veterinarian. Antibiotic treatment—ceftiofur in the majority of cases—was administered to 10 animals on Farm A (5 EXP, 5 CO) and 3 animals on Farm B (2 EXP, 1 CO). Given the small and balanced numbers across groups, concomitant antibiotic use is unlikely to have materially influenced the group comparisons; however, these animals were retained in the analysis, which is a limitation to consider when interpreting the results.

## Data Availability

The original contributions presented in the study are included in the article/supplementary material, further inquiries can be directed to the corresponding author.
